# Aloin protects against UVB-induced apoptosis by modulating integrated signaling pathways

**DOI:** 10.3389/fphar.2025.1584233

**Published:** 2025-07-11

**Authors:** Qi He, Yu-Pei Chen, Chun Wu, Hongtan Wu, Fei Li, Mingyu Li, Fangfang Chen

**Affiliations:** ^1^ The School of Public Health, Fujian Medical University, Fuzhou, China; ^2^ The School of Public Health and Medical Technology, Xiamen Medical College, Xiamen, China; ^3^ Engineering Research Center of Natural Cosmeceuticals College of Fujian Province, Xiamen Medical College, Xiamen, China; ^4^ Key Laboratory of Functional and Clinical Translational Medicine, Fujian Province University, Xiamen Medical College, Xiamen, China

**Keywords:** aloin, ROS, UVB-induced apoptosis, p38, FOXO3

## Abstract

Aloin, an anthraquinone compound, is naturally abundant in the *Aloe*. This study comprehensively investigates the photoprotective effects of aloin against UVB-induced damage in HaCaT cells, elucidating its antioxidant capacity and its role in preventing cellular apoptosis. Aloin demonstrated significant antioxidant activity in ABTS and DPPH assays, with a dose-dependent reduction in intracellular reactive oxygen species levels as evidenced by fluorescence analysis. Western blot analysis revealed that aloin inhibited the phosphorylation of both p38 and JNK, with a more pronounced effect on p38. This was further supported by IC50 values, indicating a higher inhibitory potency of aloin against p38 compared to JNK. Assessments using MTT, Hoechst, Calcein/PI staining, and flow cytometry collectively verified that aloin effectively mitigated UVB-induced apoptosis in cells. Proteomic analysis showed that aloin modulated the expression of proteins involved in critical signaling pathways, including PI3K-Akt, p53, TGF-β and pathways in cancer, promoting cell survival. Aloin upregulated proteins associated with cell cycle regulation and antioxidant responses, such as CCND3, GSTM4, GNA12, SKIL, YWHAZ, and PKN3 while downregulating pro-apoptotic protein FOXO3. These findings highlight aloin’s potential as a therapeutic agent for UVB-induced skin damage by effectively modulating cellular stress responses.

## 1 Introduction

The mechanisms of skin damage induced by ultraviolet (UV) irradiation are highly complex. The oxidative stress triggered by UV exposure can initiate inflammatory responses, accelerate cellular aging, cause DNA damage, and induce apoptosis (Dunaway et al., 2018). Oxidative stress primarily arises from the overproduction of reactive oxygen species (ROS), which react with lipids, proteins, and nucleic acids within cells and tissues, leading to cellular damage and apoptosis (Chen et al., 2021). UV-induced oxidative stress activates a cascade of signaling pathways, notably c-Jun N-terminal kinase (JNK) and p38 mitogen-activated protein kinase (p38). These kinases further activate transcription factors Activator Protein-1 (AP-1) and Nuclear Factor Kappa-Light-Chain-Enhancer of Activated B Cells (NF-κB), which are crucial regulators of cell differentiation, proliferation, and apoptosis (Chaiprasongsuk and Panich, 2022; Gülow et al., 2024). Apoptosis itself is a complex process involving the coordinated action of multiple proteins (Elmore, 2007). For instance, in the intrinsic pathway (also known as the mitochondrial pathway), the B-cell lymphoma 2 (Bcl-2)/Bcl-2-associated X protein (Bax) ratio plays a crucial role in regulating apoptosis. Bcl-2 inhibits, while Bax promotes, mitochondrial outer membrane permeabilization (Liang et al., 2000; Zhang et al., 2024). Cytochrome c release from mitochondria, upon Bax activation, forms the apoptosome with Apoptotic Protease Activating Factor 1 (APAF-1), leading to caspase activation and subsequent cell death (Dorstyn et al., 2018; Tang et al., 2021). In the extrinsic pathway, UV can directly activate Fas cell surface death receptor (Fas), leading to Fas ligand (FasL) binding and the activation of caspase-8 or -10. These initiator caspases then activate effector caspase-3, inducing apoptosis (Kamarajan and Chao, 2000; Kim et al., 2003). JNK and p38 can also directly phosphorylate the tumor-suppressor protein, p53, thereby inducing the expression of FasL to mediate apoptosis (Anwar et al., 2020). Furthermore, p53 can trigger both extrinsic and intrinsic apoptotic pathways by regulating the transcription of various pro-apoptotic genes, including Fas and Bax. Prolonged UVB exposure can also induce mutations in the p53 gene, further contributing to cellular dysfunction and apoptosis (Rodust et al., 2009).

Aloin, an anthraquinone compound and a small-molecule drug primarily found in *Aloe* species, serves as a marker for their identification (Cardarelli et al., 2017). However, high aloin concentrations can exhibit toxicity (Yang et al., 2022). Notably, aloin has been shown to induce cytotoxicity by disrupting the cell cycle, specifically causing G2/M phase arrest in Jurkat cells (Buenz, 2008). In addition, high-dose aloin administration in male F344/N Nctr rats led to abnormalities in white blood cells, neutrophils, and total cholesterol levels (Boudreau et al., 2017). Recognizing these potential adverse effects, the European Union has established a maximum limit of 0.1 mg/L for aloin content in *Aloe vera* juice intended for food and beverage consumption (Kaparakou et al., 2021). Despite its toxicity at high doses, aloin possesses diverse pharmacological properties, including anticancer, antioxidant, anti-inflammatory, anti-aging, and anti-apoptotic effects (Chang et al., 2016; Lee et al., 2019; Pan et al., 2013; Zhong et al., 2019). For instance, aloin demonstrated protective effects in a Pheochromocytoma 12 (PC12) cell model, mitigating cell death and injury induced by oxygen and glucose deprivation (OGD) (Chang et al., 2016). Notably, aloin treatment effectively normalized the expression levels of Bcl-2 and Bax mRNA, thereby preventing OGD-induced apoptosis. Furthermore, aloin has verified potential in treating combined allergic rhinitis and asthma syndrome (CARAS) by modulating the Mitogen-Activated Protein Kinase (MAPK) signaling pathway, and protecting against blood–brain barrier damage following traumatic brain injury (Feng et al., 2023). Moreover, aloin demonstrated skin-protective effects against heat-induced oxidative stress by reducing the production of interleukin-8 (IL-8), lipid peroxidation, and ROS generation, while simultaneously increasing glutathione (GSH) content and superoxide dismutase (SOD) activity (Liu et al., 2015).

Given the lack of existing research on the photoprotective potential of aloin, this study aims to investigate its ability to scavenge ROS generated by UVB irradiation in Human adult keratinocyte cell line (HaCaT) cells. Building on aloin’s well-documented antioxidant and anti-apoptotic properties, a comprehensive approach was employed to explore its mechanisms. Western blotting and kinase activity analysis against p38 and JNK were utilized to elucidate the underlying pathways. Additionally, the protective effects of aloin against UVB-induced photodamage and apoptosis in HaCaT cells were assessed using a combination of 3-(4,5-dimethylthiazol-2-yl)-2,5-diphenyltetrazolium bromide (MTT) assays, Hoechst, Calcein AM and Propidium Iodide (Calcein/PI) staining analysis, and flow cytometry. Finally, proteomic analysis was conducted to further uncover the key pathways and molecular mechanisms involved in aloin’s photoprotective effects.

## 2 Materials and methods

### 2.1 Analysis of ABTS and DPPH radical scavenging

The total antioxidant capacity of aloin was determined using the Total Antioxidant Capacity Assay Kit with ABTS (Beyotime Biotechnology Co., Ltd., Shanghai, China). Briefly, various concentrations of aloin (12.5–200 μg/mL) were incubated with the 2,2′-azino-bis(3-ethylbenzothiazoline-6-sulfonic acid) (ABTS) working solution at room temperature for 5 min. The absorbance at 734 nm was measured using a microplate reader (Infinite 200Pro, Tecan, Männedorf, Switzerland). The ABTS radical scavenging rate (%) was calculated as follows: $\left[\left(\text{OD}734\text{ of control}-\text{OD}734\text{ of Aloin}\right)/\text{OD}734\text{ of control}\right]\times 100$ . For the analysis of 2,2-diphenyl-1-picrylhydrazyl (DPPH) radical scavenging, DPPH was dissolved in anhydrous ethanol. Different concentrations of aloin (12.5–200 μg/mL) were added to the DPPH solution in the dark at room temperature for 30 min. The absorbance at 517 nm was measured using a microplate reader. The DPPH radical scavenging rate (%) was determined by the formula: $\left[\left(\text{OD}517\text{ of control}-\text{OD}517\text{ of Aloin}\right)/\text{OD}517\text{ of control}\right]\times 100$ . Trolox and ascorbic acid were employed as positive controls.

### 2.2 Analysis of HaCaT cell viability

The HaCaT cells were purchased from BeNa Culture Collection (Beijing, China). The viability of HaCaT treated with different concentrations of aloin (12.5–100 μg/mL) was assessed using MTT (BS186-5g, Biosharp, Hefei, China). HaCaT cells were cultured in Dulbecco’s Modified Eagle Medium (DMEM) supplemented with 10% fetal bovine serum (FBS) and 1% penicillin-streptomycin at 37°C in a 5% CO_2_ incubator. At 80% confluency, cells were harvested by trypsinization and plated in 96-well plates at a density of 1 × 10^5^ cells/well in 100 μL complete medium. Following a 24-h attachment period, the medium was replaced with serum-free DMEM prior to treatment. Cells were exposed to varying concentrations of aloin (with equivalent 0.5% DMSO serving as vehicle control) for either 1 or 12 h. For MTT assessment, the treatment medium was carefully removed and replaced with 90 μL serum-free DMEM plus 10 μL MTT solution (5 mg/mL in PBS), followed by 4 h of incubation at 37°C. Upon completion of incubation, the supernatant was aspirated, and 200 μL of DMSO was added to each well to dissolve the formed formazan crystals. After 10 min of shaking, the absorbance at 490 nm was measured using a microplate reader (Infinite 200Pro, Tecan).

### 2.3 ROS assay in HaCaT cells

To evaluate the effect of aloin on UVB-induced ROS generation, the ROS levels within the cells were detected using the ROS Assay Kit with CM-H2DCFDA (Beyotime Biotechnology). Initially, HaCaT cells were seeded in 96-well plates (1 × 10^5^ cells/well in 100 μL complete medium) and incubated overnight at 37°C with 5% CO_2_. Following attachment, the medium was replaced with serum-free DMEM. Cells were then pretreated with aloin (12.5–100 μg/mL) or vehicle control (0.5% DMSO) for 1 h prior to UVB exposure. UVB irradiation was performed at 312 nm (225 mJ/cm^2^) using a Bio-Sun irradiation system (Vilber Bio Imaging, France). Post-irradiation, the cells were further incubated for 1 h at 37°C in a 5% CO_2_ incubator. According to the ROS Assay Kit protocol, the DCFH-DA probe was diluted 1:1,000 in serum-free DMEM to achieve a final concentration of 10 μM. The culture medium was removed, and the cells were incubated with the diluted DCFH-DA solution for 30 min at 37°C. Upon completion of incubation, the cells were washed three times with serum-free medium to remove any unincorporated probe. Intracellular ROS levels were then measured using a fluorescence microplate reader (Infinite 200Pro, Tecan) with excitation and emission wavelengths of 488 nm and 525 nm, respectively. Additionally, fluorescence images of ROS generation were captured using a fluorescence microscope (DMi8, Leica Camera AG, Germany) to further visualize and analyze changes in intracellular ROS levels.

### 2.4 Western blot analysis

HaCaT cells were treated with different concentrations of aloin (12.5–100 μg/mL) and exposed to UVB irradiation with 312 nm (225 mJ/cm^2^). After 1 h, cells were harvested and cell lysis was performed using a sonicator (Vibra-Cell, Sonics & Materials, Inc., CT, United States) under the following conditions: 20% amplitude, 1–2 s pulses repeated 10 times with intermittent cooling to ensure complete lysis. The lysis buffer consists of 1% Triton X-100, 50 mM Tris-HCl (pH 7.4), 150 mM NaCl, 20 mM NaF, 2 mM Na_3_VO_4_, 2 mM PMSF, 0.5% glycerol, and 0.1 mM BSA, providing effective cell lysis along with protease (PMSF) and phosphatase (NaF, Na_3_VO_4_) inhibition while maintaining protein stability through glycerol and BSA. Following sonication, the lysates were centrifuged at 13,000 × *g* for 10 min at 4°C to pellet cellular debris. The resulting supernatant was carefully collected for subsequent SDS-PAGE analysis. Protein lysates were separated by 12% SDS-PAGE and transferred to a PVDF membrane at 4°C for 90 min. Following transfer, the PVDF membrane was washed three times with 1×TBST for 5 min and blocked with 5% BSA (bovine serum albumin) at room temperature for 1 h. Subsequently, the PVDF membrane was placed in a solution containing the primary antibody (diluted 1:1,000) and incubated overnight at 4°C. Primary antibodies against β-actin, p38, p-p38, JNK and FOXO3 were purchased from ABClonal (Wuhan, China), and p-JNK was purchased from Cell Signaling Technology (MA, United States). β-Actin was used as a loading control protein to normalize protein expression levels. Following incubation with the primary antibody, the diluted secondary antibody solution (diluted 1:5,000) was added, and the membrane was incubated at room temperature for 1 h. The rabbit IgG peroxidase-conjugated secondary antibody was purchased from Jackson ImmunoResearch Inc. (West Grove, PA, United States). After incubation with the secondary antibody, the membrane was washed again three times with 1×TBST. Protein bands were visualized using ECL chemiluminescence reagent (NcmECL Ultra, New Cell & Molecular Biotech Co., Ltd., Suzhou, China) and captured using the ChemiDoc™ XRS+ System equipped with Image Lab™ Software (Bio-Rad, CA, United States). The band intensity of protein expression levels was quantified using ImageJ 1.53e software.

### 2.5 Analysis of JNK1 and p38α activities

The JNK1 Kinase Enzyme System, p38α Kinase Enzyme System and ADP Glo™ Kinase Assay (Promega, WI, USA) were utilized to assess the effect of aloin on the activity of JNK1 and p38α kinases. Aloin solutions were prepared at varying concentrations. Briefly, varying concentrations of aloin (100–800 μg/mL for JNK1; 25–800 μg/mL for p38α) were added to the reaction mixture containing diluted JNK1 or p38α kinase, substrate, and ATP. The reaction was incubated at room temperature for 60 min. After incubation, the ADP-Glo™ reagent was added and incubated for an additional 60 min. Finally, kinase detection reagent was added, and the mixture was incubated for 60 min to facilitate the chemiluminescent reaction. Luminescence was measured with an integration time of 0.5–1 s using an Infinite 200Pro microplate reader (Infinite 200Pro, Tecan).

### 2.6 Analysis of HaCaT cell viability after 12 h post-UVB irradiation

HaCaT cells were cultured overnight at 37°C in a 5% CO_2_ incubator. Following attachment, the medium was replaced with serum-free DMEM. Cells were then pretreated with aloin (12.5–100 μg/mL) or vehicle control (0.5% DMSO) for 1 h prior to UVB exposure. UVB irradiation was performed at 312 nm (225 mJ/cm^2^) using a Bio-Sun irradiation system (Vilber Bio Imaging, France). Post-irradiation, the cells were further incubated for 12 h at 37°C in a 5% CO_2_ incubator. Cell viability was then assessed using the MTT assay.

### 2.7 Hoechst staining assay in HaCaT cells

Hoechst Staining Kit (Beyotime Biotechnology) was utilized to observe the cell apoptosis. HaCaT cells treated with different concentrations of aloin (12.5–100 μg/mL) were cultivated after 12 h post-UVB irradiation at a dose of 225 mJ/cm^2^. Upon completion of incubation, cells were fixed with fixative solution for 10 min. Following fixation, cells were washed twice with PBS for 3 min each. Subsequently, 0.5 mL of Hoechst 33258 staining solution was added to stain the cells for 5 min. Afterward, the staining solution was aspirated, and the cells were washed twice again with PBS for 3 min. Nuclear morphology was then observed using a fluorescence microscope (DMI8, Leica).

### 2.8 Calcein/PI staining assay in HaCaT cells

To assess cell viability and cytotoxicity, the Calcein/PI Live/Dead Viability/Cytotoxicity Assay Kit (Beyotime Biotechnology) was used. HaCaT cells treated with different concentrations of aloin (12.5–100 μg/mL) were cultivated after 12 h post-UVB irradiation at a dose of 225 mJ/cm^2^. Upon completion of incubation, the cells were stained using the Calcein/PI Kit. The culture medium was aspirated, and the cells were gently washed once with PBS. Then, according to the kit’s instructions, the Calcein/PI detection working solution was prepared and added to each well. The cells were incubated at 37°C in the dark for 30 min. After incubation, cell viability and cytotoxicity were assessed by observing fluorescence under a fluorescence microscope (DMI8, Leica).

### 2.9 Flow cytometry assay in HaCaT cells

To analyze cell apoptosis, the Annexin V-FITC Apoptosis Detection Kit (Beyotime Biotechnology) was used. HaCaT cells treated with aloin (50 μg/mL) were cultivated after 12 h post-UVB irradiation at a dose of 225 mJ/cm^2^. Upon completion of incubation, the culture medium was aspirated, and the adherent cells were washed once with PBS. Subsequently, an appropriate amount of trypsin-EDTA-free digestion solution was added, and the cells were incubated at room temperature until they could be detached. The cell suspension was then transferred to a tube and centrifuged at 1,000 × g for 5 min, with the supernatant discarded. The collected cells were gently washed by PBS, and the cells were gently resuspended in Annexin V-FITC binding buffer. Then, Annexin V-FITC and PI staining solution were added, and the mixture was gently vortexed. Cells were incubated at room temperature in the dark for 10–20 min and then placed on ice. Finally, apoptosis was analyzed using the ACEA NoVoCyte Flow Cytometer (ACEA Biosciences, Inc., San Diego, CA, United States).

### 2.10 Proteomic analysis of HaCaT cells

HaCaT cells were treated with 50 μg/mL aloin and harvested after 12 h post-UVB irradiation at a dose of 225 mJ/cm^2^. The cells were then collected for proteomic analysis, which was conducted by MajorBio Co., Ltd. (Shanghai, China). Data Independent Acquisition (DIA) methodology was employed in combination with an Orbitrap Astral mass spectrometer (Thermo Fisher Scientific, Waltham, MA, United States). Data processing, quantification, and quality assessments were carried out using proprietary software developed by MajorBio. Functional annotation of the identified proteins was performed using the UniProt database, while additional functional insights were derived from various biological databases, such as GO, KEGG, the non-redundant protein database (NR), evolutionary genealogy of genes: non-supervised orthologous groups (eggNOG), and the Search Tool for the Retrieval of Interacting Genes/Proteins (STRING). Quantitative differences between groups were assessed using Student’s t-test. Proteins showing significant differences were identified based on the criteria of absolute Log_2_ fold change (Log_2_FC) ≥ 1 and a p-value < 0.05, as performed by R analysis. GO and KEGG pathway enrichment analyses were carried out using goatools and Python, respectively. The mass spectrometry-based proteomics data have been deposited in the ProteomeXchange Consortium through the PRIDE (Perez-Riverol et al., 2025) partner repository under the dataset identifier PXD060808.

### 2.11 Data statistical analysis

Data are presented as mean ± standard deviation. Duncan’s multiple range test was used to compare means between groups, with statistical significance set at p < 0.05. All statistical analyses were performed using IBM SPSS Statistics software (SPSS Inc., Chicago, United States).

## 3 Results

### 3.1 Antioxidant capacities of aloin using ABTS and DPPH free radicals

**FIGURE 1:**
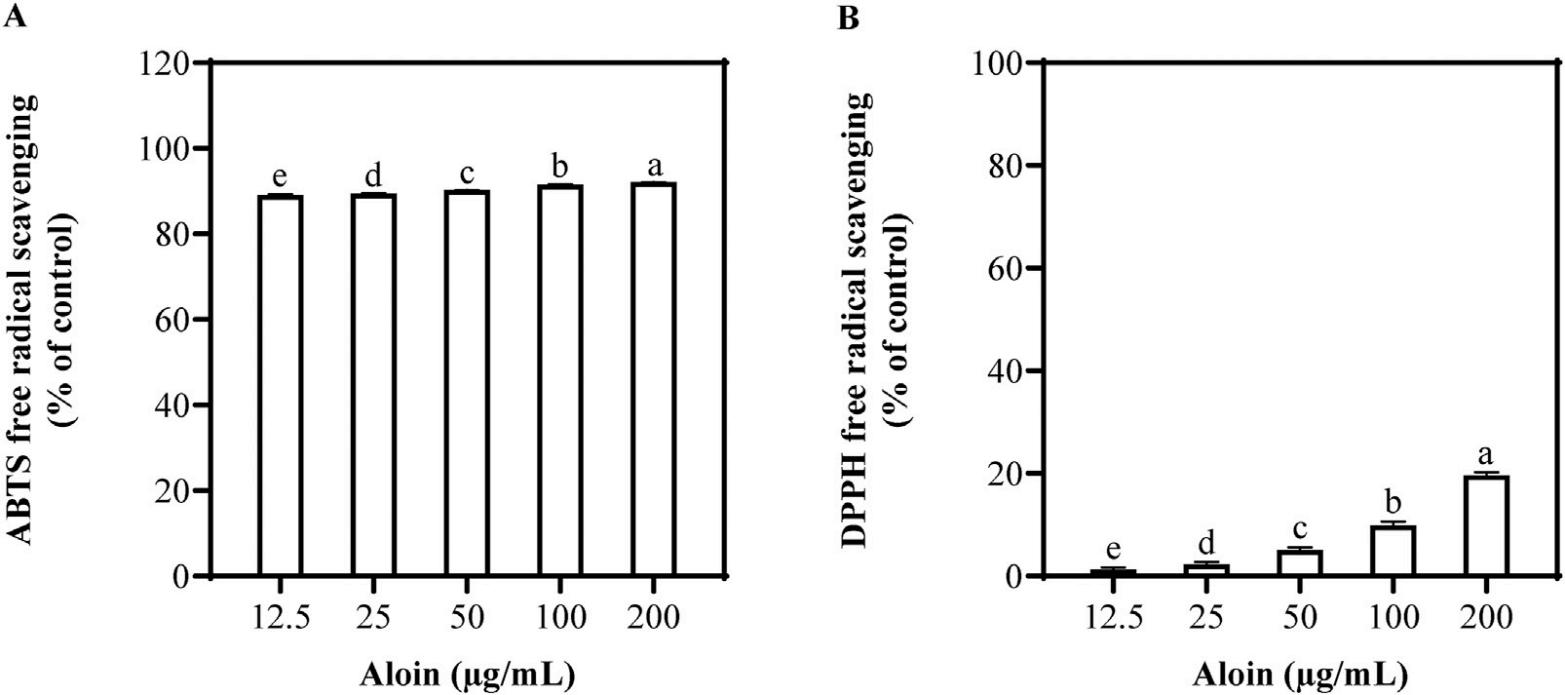
Antioxidant capacity of aloin using **(A)** ABTS and **(B)** DPPH free radical scavenging assays. DMSO was used as the control.

In the ABTS assay, aloin demonstrated remarkable efficacy (Figure 1). As its concentration increased from 12.5 μg/mL to 200 μg/mL, the scavenging rate also increased from 89.2% to 92.1%. Aloin exhibited excellent removal effects on ABTS, revealing strong antioxidant capacity. In contrast, aloin exhibited weaker scavenging activity against DPPH radicals, with a more gradual increase in scavenging rate from 1.3% at 12.5 μg/mL to 19.7% at 200 μg/mL. Trolox and ascorbic acid were employed as positive controls, with their corresponding results presented in Supplementary Figure S1.

### 3.2 HaCaT cell viability and ROS assay after 1 h post-UVB irradiation

To determine the optimal UVB irradiation dose for subsequent experiments, HaCaT cells were exposed to various UVB doses. Cell viability assays revealed no obvious impact on cell viability after 1 h post-UVB irradiation (Supplementary Figure S2). However, after 12 h post-UVB irradiation, cell viability significantly decreased with increasing UVB dose, demonstrating a dose-dependent relationship between UVB exposure and cell survival. Additionally, intracellular ROS levels increased in a dose-dependent manner after 1 h post-UVB irradiation. A UVB dose of 225 mJ/cm^2^ was selected for subsequent experiments. This dose effectively induced cellular responses while maintaining acceptable cell viability, enabling a more thorough investigation of UVB-induced cellular responses and ROS generation.

**FIGURE 2:**
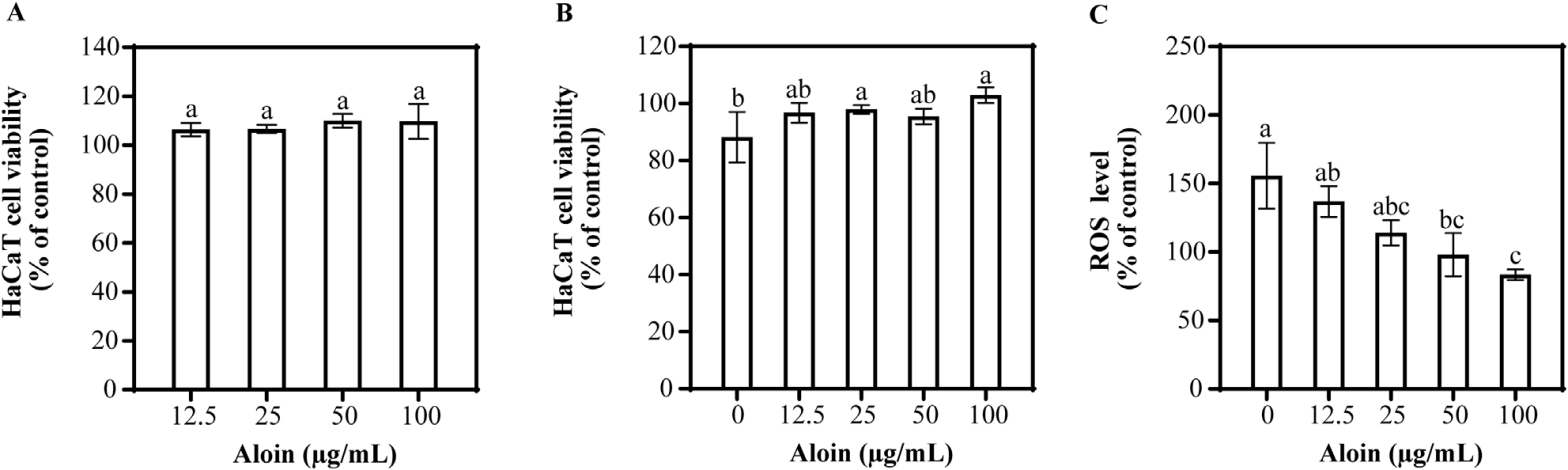
The viability of HaCaT cells treated with aloin was evaluated in the **(A)** absence and **(B)** presence of UVB irradiation (225 mJ/cm^2^) after 1 h of cultivation. **(C)** The ROS content in HaCaT cells treated with aloin was measured after 1 h post-UVB irradiation (225 mJ/cm^2^). The control remained untreated with UVB irradiation. Significance is indicated by different letters in each column (p < 0.05). Data are presented as mean ± S.D. (n = 3).

The effects of aloin on HaCaT cells with and without UVB irradiation are shown in Figure 2. Even at different concentrations, no cytotoxic effects on HaCaT cells were observed (Figure 2A). Furthermore, no significant phototoxic effects were observed in HaCaT cells treated with aloin following UVB irradiation (Figure 2B). This indicates that, in the short term, treatment with exposure to UVB radiation had no significant impact on cell viability.

To evaluate the impact of aloin on UVB-induced ROS generation in HaCaT cells, the result is shown in Figure 2C. UVB irradiation significantly increased intracellular ROS levels by 155.7% compared to the non-irradiated control. However, aloin demonstrated a significant dose-dependent reduction in UVB-induced ROS generation. At a concentration of 100 μg/mL, aloin exhibited the most potent antioxidant activity, significantly reducing ROS levels to 83.5% of the UVB-irradiated control. Quercetin, employed as a positive control, is well-established for its potent antioxidant and photoprotective properties. These effects are mediated through its protective action against UVB-induced apoptosis (Supplementary Figure S3). At concentrations ≤50 μg/mL, quercetin showed no significant effect on cell viability, regardless of UVB irradiation. Notably, treatment with quercetin for 1 h prior to UVB exposure significantly reduced intracellular ROS levels compared to the UVB-irradiated control.

**FIGURE 3:**
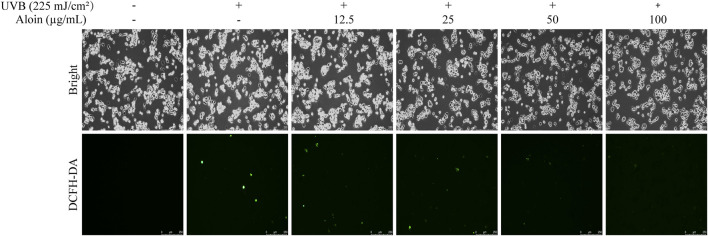
The ROS levels in HaCaT cells treated with aloin (12.5–100 μg/mL) were evaluated using fluorescence microscopy after 1 h post-UVB treatment (225 mJ/cm^2^). The scale bar in the lower right corner represents 250 μm.

The inhibitory effect of aloin on UVB irradiation-induced intracellular ROS levels was further analyzed using HaCaT cells labeled with the 2′,7′-dichlorodihydrofluorescein diacetate (DCF-DA) fluorescent probe (Figure 3). UVB irradiation significantly increased the ROS levels in cells that were not treated with aloin, leading to a marked enhancement in fluorescence intensity and causing the cells to exhibit intense green fluorescence. However, in the presence of aloin, this fluorescence intensity was notably reduced. A clear concentration-dependent reduction in ROS fluorescence intensity was observed with increasing aloin concentrations. The most pronounced inhibitory effect was observed at an aloin concentration of 100 μg/mL.

### 3.3 Analysis of Western blot and p38α and JNK1 activities

**FIGURE 4:**
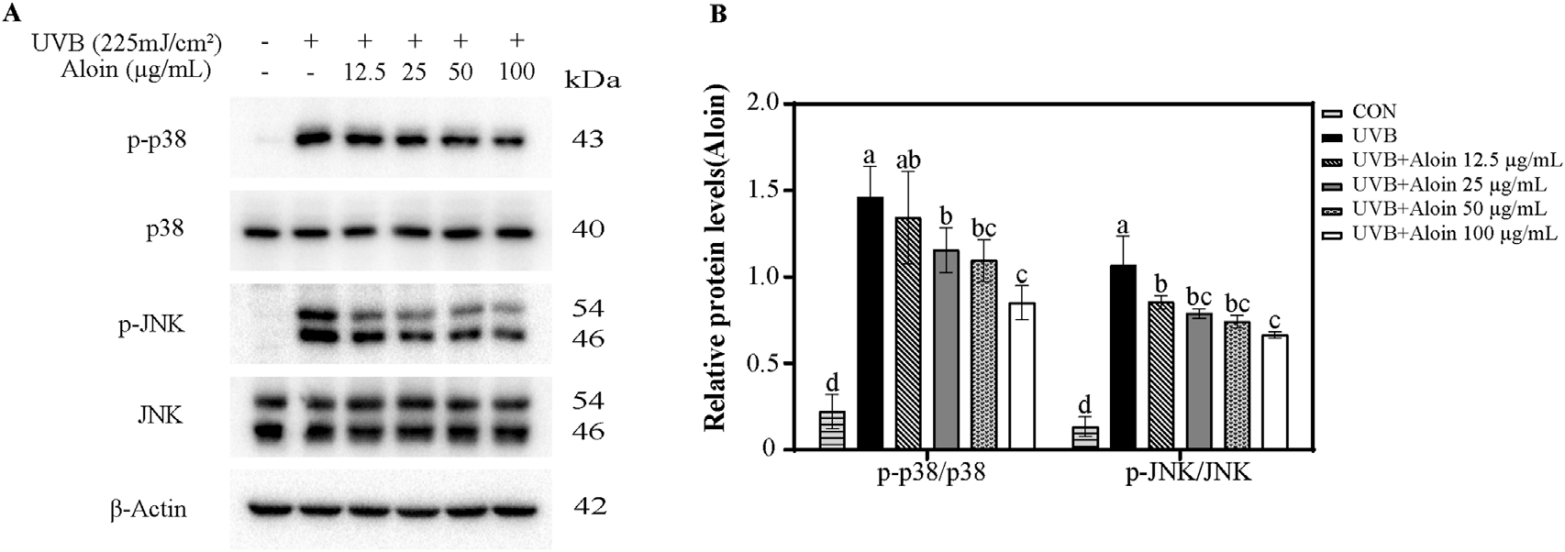
**(A)** Western blot analysis was conducted on HaCaT cells treated with aloin after 1 h post-UVB treatment (225 mJ/cm^2^). **(B)** The relative protein intensities for cells treated with aloin were quantified using ImageJ software. The control remained untreated with UVB irradiation. Statistical significance is indicated by different letters in each column (p < 0.05). Data are presented as mean ± S.D. (n = 3).

To investigate the regulatory role of aloin in UVB-induced cellular stress responses, Western blot analysis was performed to examine the phosphorylation levels of p38 and JNK proteins (Figure 4). The results revealed that UVB irradiation significantly increased the phosphorylation of both p38 and JNK, indicating activation of these signaling pathways. Meanwhile, the total levels of p38 and JNK proteins remained largely unchanged. Notably, aloin demonstrated a significant inhibitory effect on the phosphorylation of p38 and JNK. At a concentration of 100 μg/mL, aloin markedly reduced the phosphorylation ratio of p38 from 146.0% to 85.3% and that of JNK from 106.7% to 66.6%. These findings suggest that aloin effectively inhibited the activation of p38 and JNK signaling pathways, particularly p38, in response to UVB irradiation.

**FIGURE 5:**
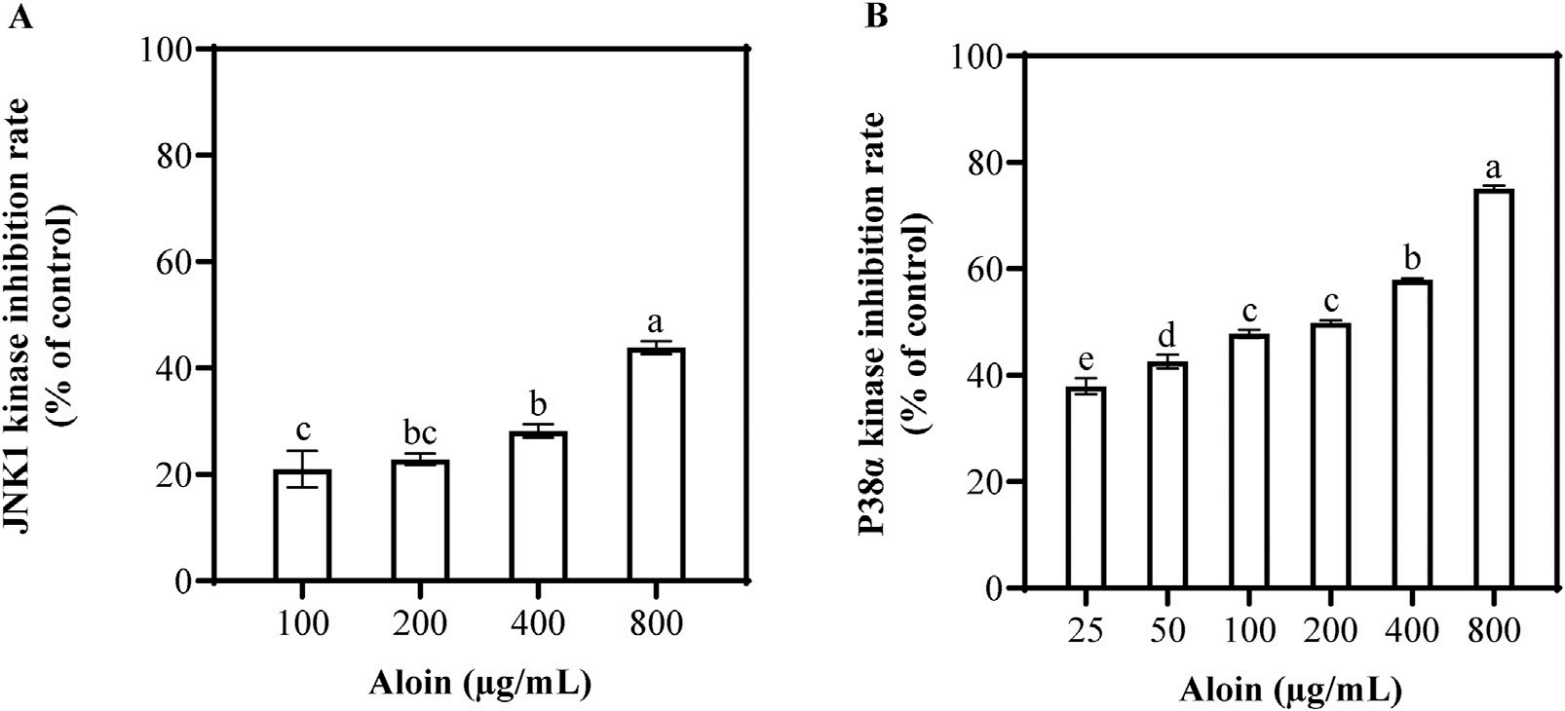
The inhibition rates of **(A)** JNK1 and **(B)** p38α kinase activities were assessed following treatment with various concentrations of aloin. Statistical significance is indicated by different letters in each column (p < 0.05). Data are presented as mean ± S.D. (n = 3).

To further elucidate the direct effects of aloin on cellular stress pathways, the activities of p38α and JNK1 kinases were assessed. The result showed that aloin significantly inhibited the activities of both kinases in a dose-dependent manner (Figure 5). Statistical analysis using SPSS revealed a more potent inhibitory effect of aloin on p38α kinase compared to JNK1 kinase. At a concentration of 800 μg/mL, aloin inhibited p38α kinase activity by 75.1%, with a half maximal inhibitory concentration (IC50) value of 93.4 μg/mL. In contrast, aloin exhibited weaker inhibition of JNK1 kinase, with an inhibition rate of 43.8% at 800 μg/mL and a higher IC50 value of 1,639.7 μg/mL. These findings further confirm the significant role of aloin in regulating cellular stress responses, particularly its direct inhibitory effect on p38α kinase.

### 3.4 Analysis of HaCaT cell viability after 12 h post-UVB irradiation

**FIGURE 6:**
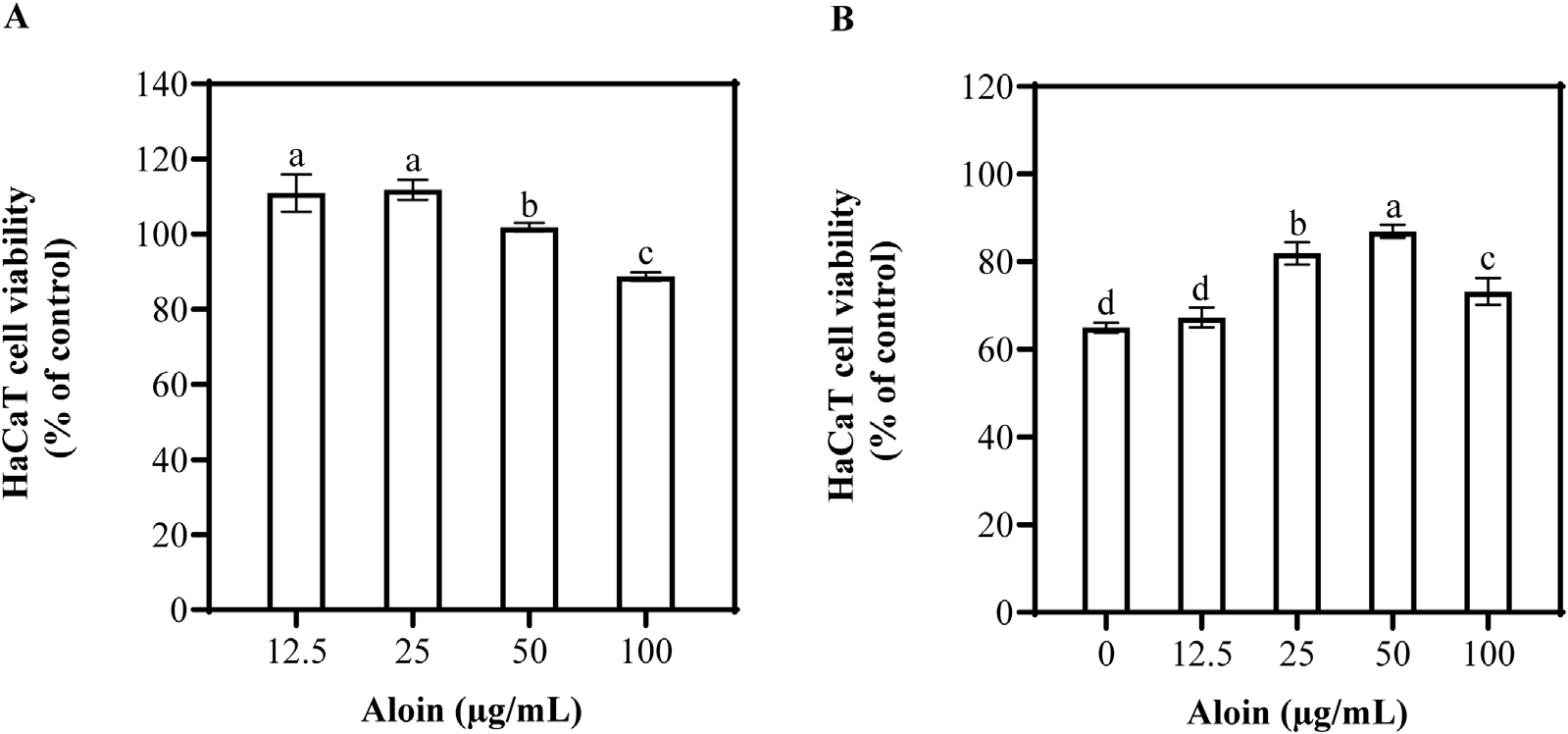
The viability of HaCaT cells treated with aloin was evaluated in the **(A)** absence and **(B)** presence of UVB irradiation (225 mJ/cm^2^) after 12 h of cultivation. Control groups consisted of **(A)** solvent control (DMSO) and **(B)** irradiation control (no UVB irradiation). Significance is indicated by different letters in each column (p < 0.05). Data are presented as mean ± S.D. (n = 3).

The MTT assay was employed to assess the impact of aloin on HaCaT cell viability. The results indicated that at a concentration of 100 μg/mL, the cell viability of aloin-treated cells was 88.7%, demonstrating a slight inhibitory effect on cell activity (Figure 6A). To further explore the protective effects of aloin against UVB-induced cellular damage, an assessment was conducted using the MTT assay. As shown in Figure 6B, aloin exhibited a certain degree of protective effect on HaCaT cells as its concentration increased. At a concentration of 50 μg/mL, aloin significantly increased cell viability to 87.0%. However, at a concentration of 100 μg/mL, while still exhibiting photoprotective effects, cell viability slightly decreased to 73.2%. As a positive control, quercetin demonstrated significant photoprotective effects by markedly improving cell viability under UVB-induced photodamage conditions (Supplementary Figure S4). At a concentration of 25 μg/mL, quercetin treatment completely restored cell viability to levels comparable to non-UVB-exposed normal cells.

**FIGURE 7:**
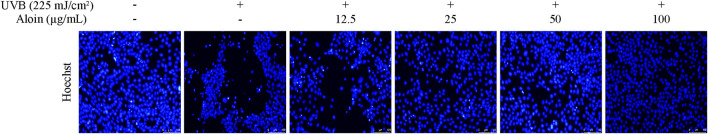
Hoechst staining was performed on HaCaT cells treated with aloin (12.5–100 μg/mL), and the results were evaluated using fluorescence microscopy after 12 h post-UVB treatment (225 mJ/cm^2^). The scale bar in the lower right corner represents 100 μm.

The protective effect of aloin on nuclear damage in HaCaT cells after UVB irradiation was evaluated through observation with Hoechst staining combined with fluorescence microscopy. Under normal conditions, cell nuclei exhibited uniform blue fluorescence. However, UVB irradiation induced nuclear damage, characterized by chromatin condensation and fragmentation, resulting in intense white fluorescence in affected cells. As the concentration of aloin gradually increased, the proportion of normal cells showed a distinct upward trend. When the concentration was increased to 50–100 μg/mL, the morphology of the cell nuclei almost completely returned to the untreated control, indicating that aloin effectively protected against UVB-induced nuclear damage (Figure 7).

**FIGURE 8:**
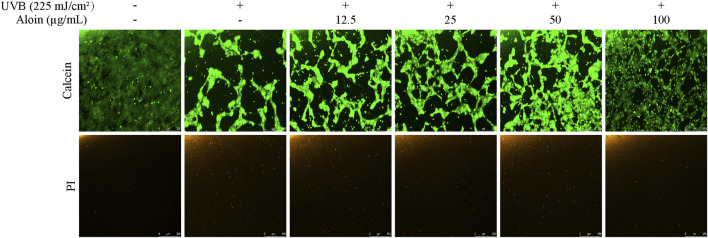
Calcein/PI staining was performed on HaCaT cells treated with aloin (12.5–100 μg/mL), and the results were evaluated using fluorescence microscopy after 12 h post-UVB treatment (225 mJ/cm^2^). The scale bar in the lower right corner represents 250 μm.

The Calcein/PI staining method was employed to assess cell viability and cytotoxicity in HaCaT cells following UVB irradiation. Living cells stained with Calcein exhibit green fluorescence, while dead cells stained with PI exhibit red fluorescence. UVB irradiation significantly decreased cell viability, as evidenced by a decrease in green fluorescence and a slight increase in red fluorescence. However, treatment with aloin significantly mitigated UVB-induced cell death. As the concentration of aloin increased, green fluorescence intensity increased, indicating an increase in the number of viable cells (Figure 8). Meanwhile, red fluorescence intensity decreased, suggesting a reduction in cell death.

**FIGURE 9:**
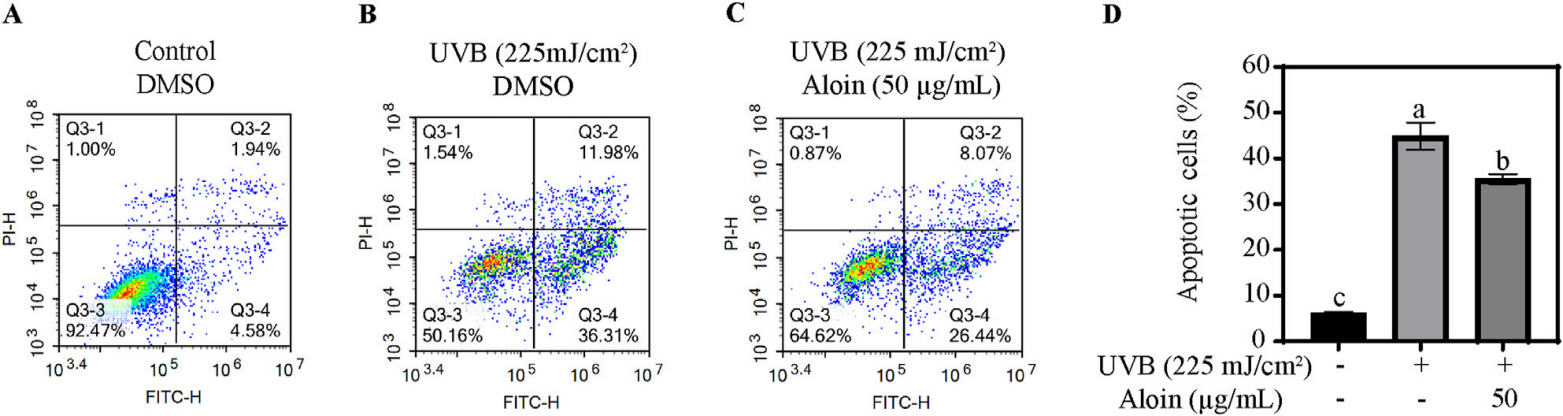
Flow cytometry analysis of HaCaT cells was performed in the **(A)** absence and **(B)** presence of UVB treatment (225 mJ/cm^2^) after 12 h of cultivation. HaCaT cells treated with **(C)** aloin was assessed using flow cytometry after 12 h post-UVB treatment (225 mJ/cm^2^). The flow cytometry results are presented as representative diagrams. **(D)** Apoptotic cells were quantified in quadrants Q3-2 and Q3-4. Significance is indicated by different letters in each column (*p* < 0.05). Data are presented as mean ± S.D. (n = 3).

The impact of UVB irradiation on HaCaT cell viability and apoptosis, as well as the protective role of aloin, was investigated using flow cytometry and Annexin V-FITC/PI staining. In the control group, without UVB irradiation, cell viability averaged 93.0%, with low apoptosis (6.2%) and necrosis rates (0.8%) (Figure 9). However, UVB irradiation significantly decreased cell viability to 53.7%, while concomitantly increasing the apoptosis rate to 44.9% and also elevating the necrosis rate. On the other hand, treatment with 50 μg/mL aloin significantly mitigated UVB-induced damage. Cell viability increased to 63.8%, and the apoptosis rate decreased to 35.5%. While the necrosis rate remained largely unchanged, these findings demonstrate the protective effect of aloin against UVB-induced cell death.

### 3.5 Proteomic analysis of HaCaT cell after 12 h post-UVB irradiation

The proteomic effect of 50 μg/mL aloin on HaCaT cell damage induced by UVB was evaluated through comparative protein expression. Compared to control cells, UVB irradiation significantly altered protein expression in HaCaT cells, with 64 proteins upregulated and 236 proteins downregulated (Supplementary Figure S5). In cells treated with both UVB and aloin, 79 proteins were upregulated and 84 proteins were downregulated compared to the UVB-only group. Analysis of the heatmap analysis revealed distinct protein expression patterns among the three groups. Supplementary Tables S1, S2 list the top 20 most significantly upregulated and downregulated proteins, respectively.

Functional annotations of differentially expressed proteins were conducted using the Gene Ontology (GO) and Kyoto Encyclopedia of Genes and Genomes (KEGG) databases (Supplementary Figure S6). In the aloin-treated group, differentially expressed proteins exhibited significant enrichment across multiple dimensions, including Biological process, Molecular function, and Cellular component. In the GO functional enrichment analysis, these proteins were mainly enriched in key pathways such as “Signal transduction,” “Intracellular signal transduction” and “MAPK cascade” (Supplementary Figure S7). This indicates that aloin exerted its protective effects on cells through mechanisms involving the regulation of signaling networks and cell differentiation. In the KEGG functional enrichment analysis (Supplementary Figure S8), differentially expressed proteins in the aloin-treated group were primarily enriched in several crucial pathways, including “Pathways in cancer,” “TGF-beta signaling pathway,” “Chemical carcinogenesis,” and “p53 signaling pathway” (Table 1). Within the p53 signaling pathway and pathways in cancer, key proteins such as G1/S-specific cyclin-D3 (CCND3), Glutathione S-transferase Mu 4 (GSTM4), and G protein subunit alpha 12 (GNA12) were significantly upregulated. This suggests that aloin may enhance cellular resistance to UVB damage by regulating cell cycle progression and promoting antioxidant responses. Furthermore, the significant increase in Ski-like protein (SKIL) expression within the TGF-beta signaling pathway further supports aloin’s role in cellular repair. Another finding is that some proteins within the Ras-related protein 1 (Rap1) signaling pathway, such as Rap guanine nucleotide exchange factor 5 isoform 1 (RAPGEF5) and RAP1 GTPase activating protein (RAP1GAP), were significantly downregulated following UVB stress.

**TABLE 1:** KEGG enrichment analysis of differentially expressed proteins compared with aloin and DMSO control.

KEGG description	Gene	Protein name	FC	Log_2_FC	p value
p53 signaling pathway	CCND3	G1/S-specific cyclin-D3	32	5	4.33E-06
Pathways in cancer	CCND3	G1/S-specific cyclin-D3	32	5	4.33E-06
	GSTM4	Glutathione S-transferase Mu 4	32	5	1.44E-07
	GNA12	G protein subunit alpha 12	32	5	5.65E-10
TGF-β signaling pathway	SKIL	Ski-like protein	32	5	2.89E-08
Rap1 signaling pathway	RAPGEF5	Rap guanine nucleotide exchange factor 5 isoform 1	1.00E-05	−16.61	5.70E-07
	RAP1GAP	RAP1 GTPase activating protein	1.00E-05	−16.61	4.46E-07

Proteomic analysis revealed significant alterations in the expression of key proteins within the Phosphoinositide 3-Kinase/Protein Kinase B (PI3K-Akt) signaling pathway in the aloin-treated group. Specifically, the expression of CCND3, Tyrosine 3-monooxygenase (YWHAZ), Myc proto-oncogene protein (MYC), Collagen alpha-2(VI) chain (COL6A2), and Serine/threonine-protein kinase N3 (PKN3) was significantly upregulated (Table 2). Conversely, the expression of Forkhead box protein O3 (FOXO3), a key pro-apoptotic protein, was significantly downregulated. These findings suggest that aloin effectively protected cells from UVB-induced damage by modulating the PI3K-Akt signaling pathway, inhibiting apoptotic signals, and promoting cell cycle progression and survival.

**TABLE 2:** Differentially expressed proteins in the PI3K/Akt pathway compared with aloin and DMSO control.

Gene	Protein name	FC	Log_2_FC	p value
CCND3	G1/S-specific cyclin-D3	32	5	4.33E-06
YWHAZ	Tyrosine 3-monooxygenase	32	5	2.60E-08
FOXO3	Forkhead box protein O3	1.00E-05	−16.61	3.22E-05
MYC	Myc proto-oncogene protein	2.75	1.46	2.212E-4
COL6A2	Collagen alpha-2(VI) chain	32	5	5.09E-08
PKN3	Serine/threonine-protein kinase N3	32	5	1.867E-4

Utilizing the STRING database, we employed network modeling to construct a protein-protein interaction (PPI) network, which enabled us to systematically identify critical interactions from complex biological data (Supplementary Figure S9). Our findings reveal that treatment with aloin significantly upregulated the expression of DUSP6 (encoding Dual-specificity protein phosphatase) and CDT1 (encoding DNA replication factor Cdt1), with both proteins exhibiting a Log_2_ fold change (FC) of 5. Through these two proteins, aloin effectively downregulated the MAPK signaling pathway and promoted DNA repair following UVB damage. This coordinated regulation highlights the protective role of DUSP6 and CDT1 against UVB-induced cellular stress.

Western blot analysis was performed to validate FOXO3, a critical regulator identified through our proteomic screening. As shown in Supplementary Figure S10, the protein levels of FOXO3 were significantly reduced in the aloin-treated group compared to the UVB-only group. This finding confirms the results obtained from our proteomic analysis.

## 4 Discussion

Aloin, as an antioxidant, demonstrated varying scavenging capacities against different types of free radicals. While exhibiting significant removal of ABTS radicals, its activity against DPPH radicals was relatively weaker (Figure 1). However, aloin displayed a dose-dependent scavenging effect on DPPH radicals. As the aloin concentration increased from 12.5 μg/mL to 200 μg/mL, the DPPH scavenging rate gradually rose, reaching a maximum of 19.7%. These findings align with previous research by Mohamed (2014), who reported DPPH scavenging activity by aloin at a concentration of 20 mM (equivalent to 8,367.8 μg/mL), with a maximum inhibition rate of 36.2% (Mohamed, 2014). Although the scavenging effect of aloin on DPPH radicals was limited, its antioxidant activity significantly increased with rising concentrations.

Aloin exhibits UV-absorbing properties with maximum absorption peaks at 266, 298, and 354 nm (Nagaraju et al., 2011). Additionally, this study demonstrated a dose-dependent reduction in UVB-induced ROS levels with increasing aloin concentrations. At 100 μg/mL, aloin significantly reduced ROS levels to 83.5%, compared to a 155.7% increase in the UVB-irradiated control (Figure 2). These findings align with previous studies demonstrating aloin’s antioxidant activity in various oxidative stress models. For instance, Ma et al. (2018) reported that aloin mitigates oxidative stress by inhibiting LPS-induced ROS generation, thereby exerting anti-inflammatory effects (Ma et al., 2018). Similarly, Jing et al. (2020) demonstrated aloin’s protective effects in a traumatic brain injury model, where it protected the blood-brain barrier by reducing ROS generation (Jing et al., 2020). Moreover, Chang et al. (2016) demonstrated that aloin significantly reduced ROS and calcium ion overload caused by OGD-reperfusion, enhanced mitochondrial membrane potential (MMP), and thereby protected mitochondrial function (Chang et al., 2016). Liu et al. (2015) found that aloin protected skin fibroblasts from heat stress damage by reducing excessive ROS generation, increasing intracellular GSH levels, and enhancing SOD activity (Liu et al., 2015). Pengjam et al. found that aloin significantly decreased receptor of the NF-κB ligand (RANKL)-induced ROS generation, inhibited excessive ROS production, and effectively prevented osteoclast differentiation, thereby suppressing bone resorption (Pengjam et al., 2016). Furthermore, aloin can effectively reduce oxidative stress markers such as hydrogen peroxide and lipid peroxidation induced by UVB (Silva et al., 2014). These findings collectively support the potential of aloin as a natural antioxidant in mitigating UVB-induced oxidative damage.

Given that UVB significantly activates the p38 and JNK signaling pathways, this study observed that treatment with 100 μg/mL aloin markedly inhibited the phosphorylation levels of p38 and JNK induced by UVB (Figure 4). This finding aligns with previous research demonstrating the inhibitory effects of aloin on these signaling pathways. Zhong et al. (2019) showed that aloin alleviated D-galactose-induced cognitive impairment and inflammation in mice by downregulating p38 and JNK signaling pathways (Zhong et al., 2019). Additionally, aloin has been shown to inhibit LPS-induced inflammation and apoptosis by suppressing upstream kinases, such as p38, within the NF-κB signaling pathway (Luo et al., 2018). In contrast, Na et al. (2016) reported that IL-1β significantly stimulated the production of IL-8 in epithelial cells by activating the p38 and extracellular signal-regulated kinase (ERK) signaling pathways, thereby exacerbating inflammatory responses (Na et al., 2016). Nevertheless, aloin effectively reduced IL-1β-induced IL-8 production by inhibiting the activation of these signaling pathways, thus exerting anti-inflammatory effects. According to the study by Van Laethem et al. (2004), p38 MAPK induces apoptosis by promoting Bax translocation to the mitochondria, leading to cytochrome c release and caspase activation (Van Laethem et al., 2004). Inhibiting the p38 MAPK signaling pathway can effectively protect cells from UVB-induced damage, reducing the risk of UVB-induced skin damage and carcinogenesis. Jing et al. (2020) demonstrated that aloin exerts its anti-inflammatory and protective effects through inhibiting the phosphorylation of p38, thereby alleviating neuronal damage and blood-brain barrier disruption (Jing et al., 2020). Therefore, the development of p38 or JNK inhibitors has also become a potential strategy for addressing related diseases.

This study further evaluated the inhibitory effects of aloin on p38 and JNK1 kinases. Aloin demonstrated dose-dependent inhibition of p38α and JNK1 kinase activity (Figure 5). Compared to known p38 inhibitors such as VPC00628 (IC50 = 7 nM) and PH-797804 (IC50 = 16.4 nM), aloin exhibited a lower potency in inhibiting p38 kinase activity (IC50 = 223 nM), although it was more potent than 1,3,4-triarylpyrazole derivative (IC50 = 515 nM) and CBS-3595 (IC50 = 500 nM) (Madkour et al., 2021). Similarly, aloin’s inhibitory effect on JNK1 kinase was weaker compared to known inhibitors such as AS601245 (IC50 = 150 nM) and AS602801 (IC50 = 80 nM) (Wu et al., 2020). According to the study by Feng et al. (2023), molecular docking results indicated a strong binding affinity between aloin and JNK, with a binding energy of −7.4 kcal/mol, suggesting potential modulation of the MAPK8 pathway (Feng et al., 2023). Our results also showed that the binding energy between aloin and JNK1 was −7.4 kcal/mol, with two hydrogen bonds formed. Moreover, the binding energy between aloin and p38α protein was −8.5 kcal/mol, with a critical hydrogen bond formed between aloin and the tyrosine 35 (TYR35) residue (Supplementary Figure S11). This finding further confirmed the favorable binding affinity between aloin and p38α, which was consistent with the kinase inhibition rates observed in this study. The effects of aloin on HaCaT cells may also influence the phosphorylation levels of p38 and JNK themselves. As evidenced by Western blot analysis, the phosphorylation levels of these proteins decreased with increasing concentrations of aloin (Figure 4).

Based on MTT, Hoechst, Calcein/PI staining, and flow cytometry assays, aloin significantly protected cells from UVB-induced apoptosis (Figures 6–9). In proteomics analysis, a marked upregulation of FOXO3 in HaCaT cells following UVB exposure was observed. However, upon treatment with aloin, the expression of FOXO3 was significantly downregulated, effectively preventing UVB-induced apoptosis (Table 2; Supplementary Table S2). This suggests that aloin may exert its pro-proliferative and anti-apoptotic effects by modulating the p38α MAPK and FOXO3 pathways. According to the study by Ho et al. (2012), p38 regulates the translocation of FOXO3 from the cytoplasm to the nucleus by phosphorylating the Ser-7 site of FOXO3 (Ho et al., 2012). The nuclear localization of FOXO3 is closely associated with the regulation of biological processes such as the cell proliferation and apoptosis. Thus, as demonstrated by Zheng et al. (2014), berberine (BBR) activates the p38α MAPK signaling pathway, upregulates FOXO3 and p53, and inhibits the proliferation of non-small cell lung cancer (NSCLC) cells while inducing cell cycle arrest (Zheng et al., 2014). Therefore, we speculate that aloin may inhibit the nuclear translocation of FOXO3 by suppressing the p38 signaling pathway, thereby affecting cell cycle progression and apoptotic responses. Additionally, in HaCaT cells exposed to UVB, no expression of tyrosine 3-monooxygenase (YWHAZ) was detected. However, aloin treatment significantly upregulated the expression of YWHAZ (14-3-3ζ). YWHAZ is known to regulate the nucleo-cytoplasmic translocation of FOXO3 by binding to its phosphorylated form, thereby influencing its transcriptional activity (Bernardo et al., 2023). During stress responses, YWHAZ plays a crucial role in cellular adaptation to oxidative stress by modulating FOXO3 activity. DUSP6, a dual-specificity phosphatase, negatively regulates the MAPK signaling pathway (Ramkissoon et al., 2019). Aloin appeared to upregulate DUSP6 expression, thereby promoting JNK dephosphorylation and protecting cells from UVB-induced damage. This effect was further supported by Western blot analysis of p-JNK levels, which showed a dose-dependent decrease in JNK phosphorylation with increasing aloin treatment (Figure 4). These findings suggest that aloin may exert its protective effects by regulating the interplay between p38α MAPK, FOXO3, YWHAZ, DUSP6 and JNK in response to UVB-induced stress.

In other PI3K-AKT signaling pathways, aloin treatment significantly upregulated the expression of COL6A2, CCND3, MYC, and PKN3. Aloin may promote the expression of COL6A2 to aid in the repair of the extracellular matrix within the skin. The α2(VI) chain, encoded by COL6A2, is a crucial component of the extracellular matrix, widely distributed in skin, muscle, and connective tissues. It plays a critical role in maintaining the stability of the extracellular matrix and the integrity of tissue structure (Bönnemann, 2011; Fitzgerald et al., 2013). UVB irradiation can disrupt the function of collagen VI, potentially contributing to skin damage. Therefore, aloin’s upregulation of COL6A2 expression may contribute to the restoration of extracellular matrix stability and the repair of UVB-induced skin damage. On the other hand, MYC promotes cancer cell proliferation and inhibits apoptosis by regulating multiple components of the PI3K/AKT/mTOR pathway (Rebello et al., 2017). Moreover, CCND3 functions as a key regulator in driving cell proliferation and avoiding apoptosis (Ketzer et al., 2022). CDT1, a critical DNA replication licensing factor, is recruited to DNA damage sites during damage-induced repair synthesis, where it interacts with PCNA (proliferating cell nuclear antigen) to facilitate DNA repair processes (Zhang, 2021). Both CDT1 and CCND3 play key roles in cell cycle regulation. Aloin may promote post-UVB DNA repair by upregulating CDT1 and CCND3 through their synergistic interplay (Ding et al., 2012). PKN3 expression is regulated by the PI3K signaling pathway, specifically through the catalytic subunit p110β, contributing to angiogenesis and invasive cancer cell behavior (Leenders et al., 2004; Mukai et al., 2016). These results suggest that aloin may promote cell proliferation and prevent apoptosis by modulating the PI3K-AKT signaling pathway.

Additionally, in our proteomic KEGG enrichment analysis, the expression of GSTM4 in cells was significantly upregulated following treatment with aloin (Table 1). GSTM4 is an important member of the GST superfamily (Bogdani et al., 2013). By conjugation to reduced glutathione, GSTM4 helps maintain the intracellular redox state, thereby inhibiting oxidative stress-induced cell damage and apoptosis. Studies have also shown that GSTM4 may enhance the expression of antioxidant genes by interacting with the nuclear factor erythroid 2-related factor 2 (Nrf2) signaling pathway, further strengthening the cell’s antioxidant capacity (Liu et al., 2023). Furthermore, GNA12, another upregulated protein, interacts with RAS p21 protein activator 2 (RASA2) to inhibit HRas proto-oncogene (HRAS), thereby activating the PI3K/Akt/mTOR signaling pathway (Gismondi et al., 2021). This pathway promotes cell growth and inhibits apoptosis, thus enhancing cell survival. Additionally, GNA12 also activates Ras homolog gene family guanine nucleotide exchange factor (RhoGEF) and Ras homolog gene family member A (RhoA), which in turn regulate Rho-associated coiled-coil containing protein kinase (ROCK) activity and subsequently influence mTOR signaling, profoundly impacting cell growth and metabolism.

UV irradiation activates the TGF-β signaling pathway, leading to the excessive secretion of matrix metalloproteinases (MMPs) and pro-inflammatory cytokines, consequently causing collagen degradation and structural damage to the skin (Ke and Wang, 2021). Ski, a known negative regulator of the TGF-β/Smad signaling pathway, interacts with Smad proteins to inhibit their activity, thereby influencing cell proliferation, differentiation, and apoptosis (Miyazono et al., 2003; Tecalco-Cruz et al., 2018). Our proteomics analysis revealed that SKIL expression was undetectable in HaCaT cells following UVB irradiation. However, upon treatment with aloin, SKIL expression was significantly upregulated. This finding suggests that aloin may exert its anti-apoptotic effects by modulating the TGF-β/Smad signaling pathway through the upregulation of SKIL. Finally, guanine nucleotide exchange factors (GEFs) are crucial for activating Rap1 by promoting the exchange of GDP for GTP. Activated Rap1 can inhibit downstream effectors of Ras, thereby indirectly suppressing p38 activation and modulating cellular responses to stress signals (Hattori and Minato, 2003; Stork and Dillon, 2005). Our proteomic analysis revealed downregulation of both RAPGEF5 and RAP1GAP. This suggests that the activation and inactivation of Rap1 may be disrupted, leading to a low-activity or dormant state of the Rap1 signaling pathway. Therefore, we speculate that aloin may not exert its effects through the Rap1 signaling pathway.

Aloin exerted comprehensive protection against UVB-induced cellular damage through a coordinated network of molecular mechanisms that synergistically target oxidative stress, DNA repair, survival signaling, and inflammatory responses. As a potent antioxidant, aloin demonstrates dose-dependent free radical scavenging activity, reducing UVB-induced ROS levels while upregulating the glutathione conjugation enzyme GSTM4 to enhance cellular antioxidant capacity. Aloin could directly modulate key stress-response pathways by inhibiting p38α kinase activity through the high molecular interaction (binding energy of −8.5 kcal/mol), while simultaneously upregulating the dual-specificity phosphatase DUSP6 to promote JNK dephosphorylation. This kinase inhibition disrupts the p38-FOXO3 apoptotic axis, reducing nuclear FOXO3 translocation and subsequent activation of pro-apoptotic genes. Concurrently, aloin activated survival pathways by upregulating PI3K-AKT components including CCND3, MYC and PKN3, while stimulating extracellular matrix repair through COL6A2 overexpression. The DNA damage response was enhanced via recruitment of the licensing factor CDT1 to damage sites for PCNA-mediated repair synthesis. Aloin further demonstrated anti-inflammatory effects by upregulating the TGF-β inhibitor SKIL, reducing MMP secretion and inflammatory cytokine production. This multi-targeted approach, simultaneously addressing oxidative damage (ROS scavenging), genotoxic stress (through CDT1/PCNA-mediated repair), apoptotic triggers (via FOXO3/YWAHZ regulation), and inflammatory responses (through SKIL/TGF-β modulation), explained aloin’s superior cytoprotective efficacy compared to single-pathway targeted agents.

## 5 Conclusion

Aloin exhibited significant protective effects against UVB-induced apoptosis. Firstly, aloin demonstrated UV-absorbing properties and effectively scavenged free radicals and reactive oxygen species (ROS). Secondly, our findings, supported by molecular docking experiments, revealed that aloin mildly inhibits p38 protein activity. Western blot analysis further confirmed that aloin reduced the phosphorylation levels of p38 and JNK, leading to the downregulation of downstream FOXO3 protein. Furthermore, proteomic analysis revealed that aloin modulated the expression of key proteins across multiple signaling pathways, including p53, pathways in cancer, and the TGF-β signaling pathway. These modulations effectively prevented apoptosis and promoted cell proliferation. Therefore, aloin’s anti-apoptotic effects were likely mediated through a multi-pronged mechanism, involving the coordinated action of multiple signaling pathways. This study provides a comprehensive mechanistic understanding of aloin’s role in promoting cellular antioxidant activity and preventing UVB-induced cellular damage at the protein level, offering robust evidence for its potential applications in dermatological therapeutics.

## Data Availability

The datasets presented in this study can be found in online repositories. The names of the repository/repositories and accession number(s) can be found in the article/Supplementary Material.
