# Supplementary material for: Aloin protects against UVB-induced apoptosis by modulating integrated signaling pathways
Source: Front Pharmacol. 2025 Jul 11;16:1584233. doi: 10.3389/fphar.2025.1584233 (PMC12289626; doi:10.3389/fphar.2025.1584233)
Supplement: Supplementary file 1 [file DataSheet1.docx]

Supplementary Material





**Supplementary Figure 1.** Antioxidant capacity of (A) Trolox and (B) ascorbic acid using ABTS and DPPH free radical scavenging assays.





**Supplementary Figure 2.** HaCaT cell viability after treatment with various doses of UVB was assessed after (A) 1 h and (B) 12 h of cultivation. (C) ROS levels of HaCaT cell treated with various doses of UVB were assessed after 1 h cultivation. Different letters above the columns indicate statistically significant differences (p < 0.05). Data are presented as mean ± S.D. (n = 3).

**

**

**Supplementary Figure 3.** The viability of HaCaT cells treated with quercetin was evaluated in the (A) absence and (B) presence of UVB irradiation (225 mJ/cm²) after 1 h of cultivation. (C) The ROS content in HaCaT cells treated with quercetin was measured after 1 h post-UVB irradiation (225 mJ/cm²). The control remained untreated with UVB irradiation. Significance is indicated by different letters in each column (p < 0.05). Data are presented as mean ± S.D. (n = 3).

**

**

**Supplementary Figure 4.** The viability of HaCaT cells treated with quercetin was evaluated in the (A) absence and (B) presence of UVB irradiation (225 mJ/cm²) after 12 h of cultivation. Significance is indicated by different letters in each column (p < 0.05). Data are presented as mean ± S.D. (n = 3).


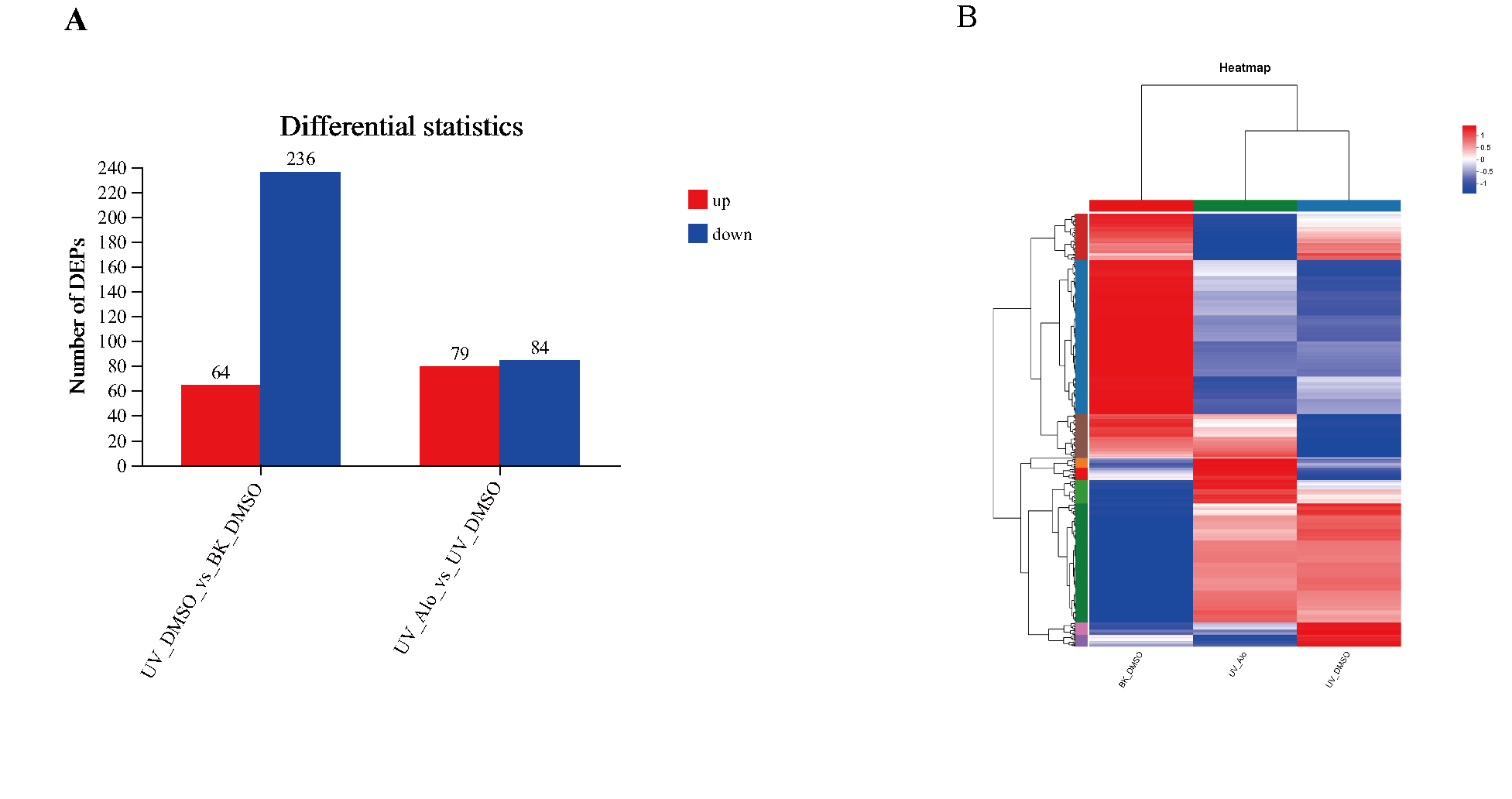


**Supplementary Figure 5.** (A) The statistics and (B) heatmap cluster of differentially expressed proteins treated with aloin. UV_DMSO and BK_DMSO indicate HaCaT cells in the presence and absence of UVB exposure (225 mJ/cm²) after 12 h of cultivation, respectively. UV_Alo indicates HaCaT cells treated with 50 μg/mL aloin after 12 h post-UVB treatment (225 mJ/cm²).


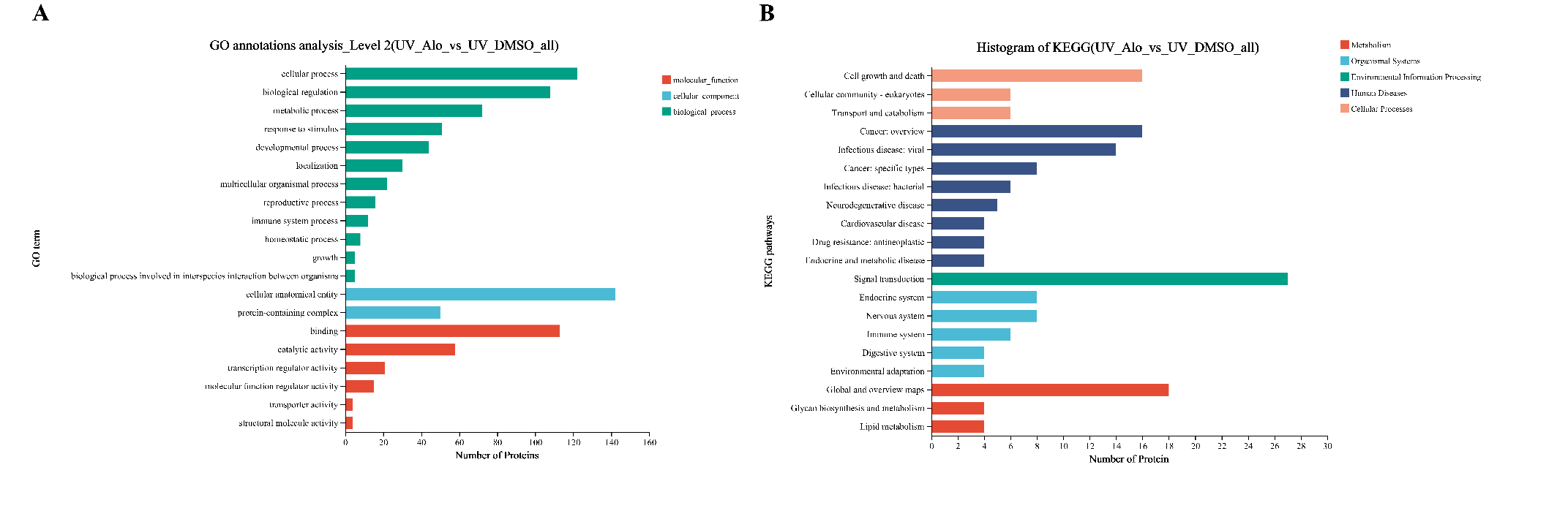
**Supplementary Figure 6.** (A) GO and (B) KEGG annotation of differentially expressed proteins compared with aloin and DMSO control.


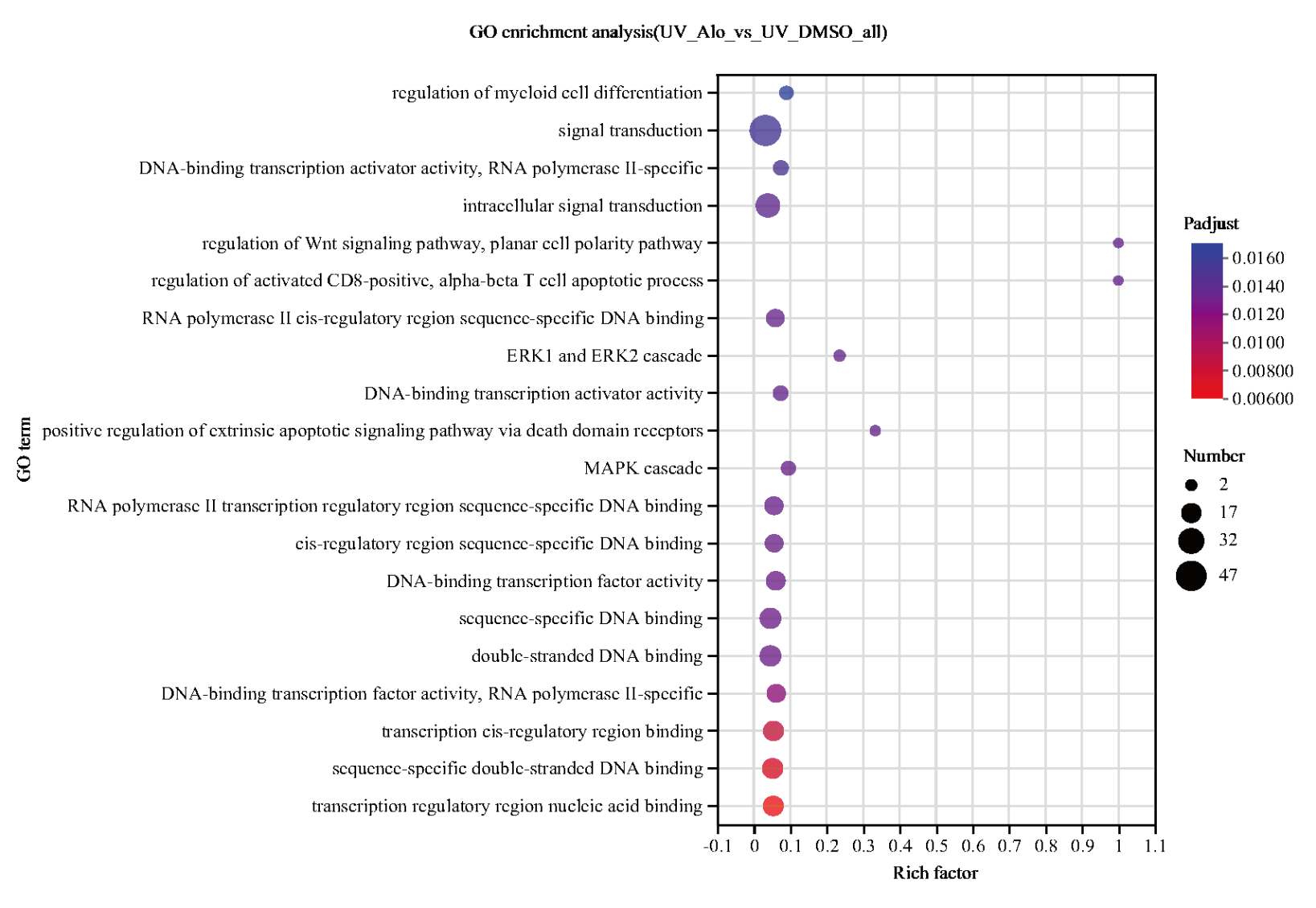


**Supplementary Figure 7.** GO enrichment analysis of differentially expressed proteins compared with aloin and DMSO control.


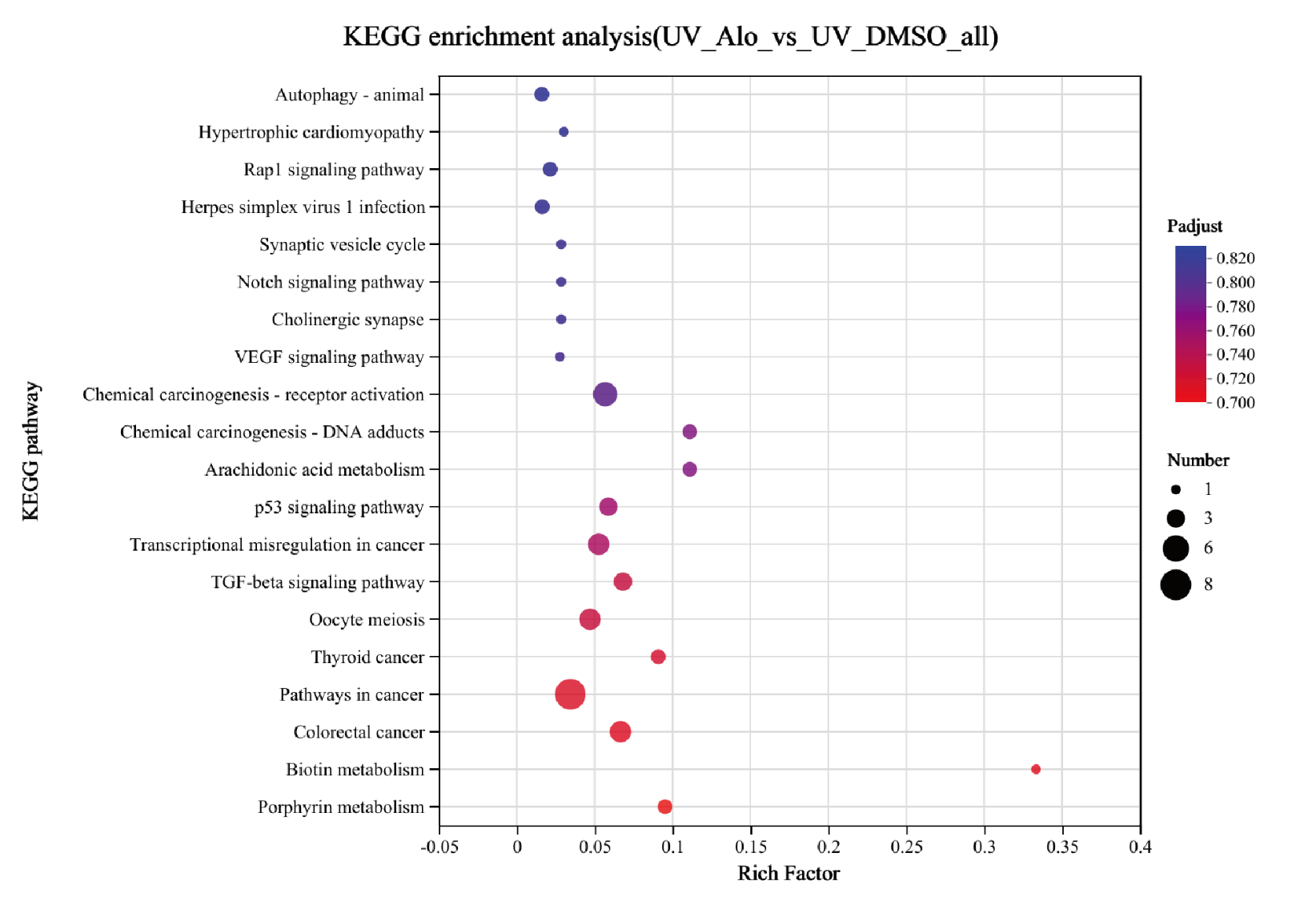


**Supplementary Figure 8.** KEGG enrichment analysis of differentially expressed proteins compared with aloin and DMSO control.


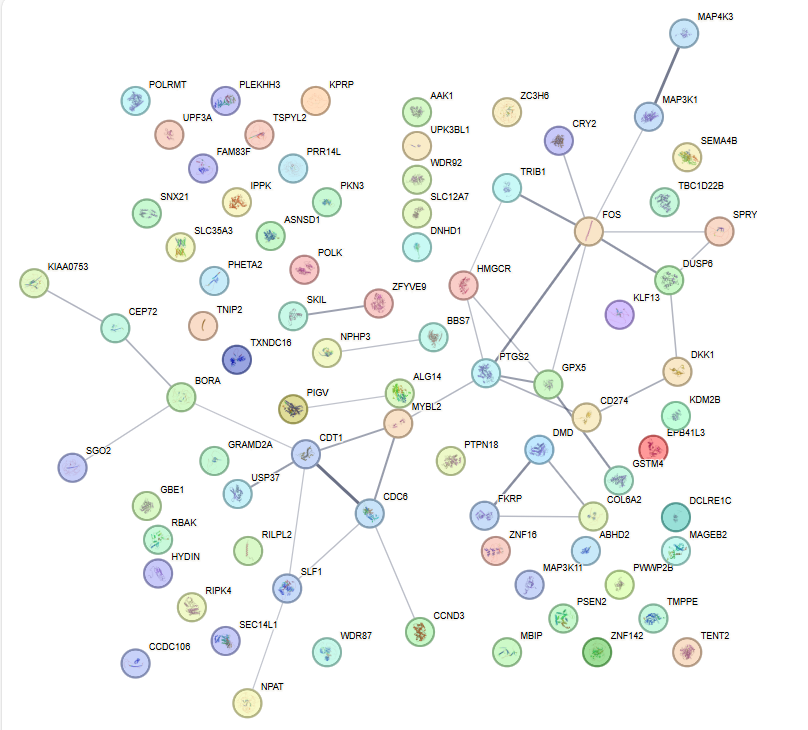


**Supplementary Figure 9.** Protein-protein interaction (PPI) network of differentially expressed proteins compared with aloin and DMSO control.





**Supplementary Figure 10.** Western blot analysis was conducted on HaCaT cells treated with aloin (50 μg/mL) after 12 h post-UVB treatment (225 mJ/cm²).


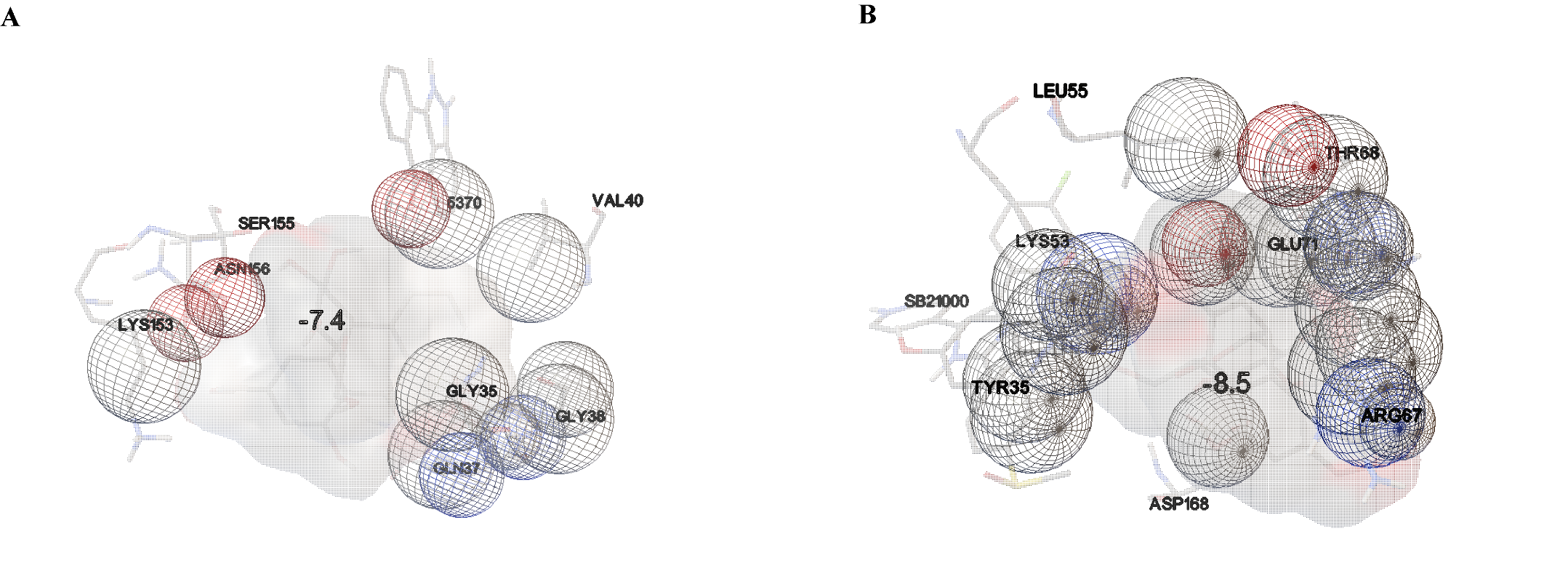


**Supplementary Figure 11.** The molecular docking of aloin with (A) JNK1 and (B) p38α enzymes. Enzymatic structures of JNK1 (1UKI) and p38α (3ZS5) from the RCSB Protein Data Bank (https://www.pdbus.org/) were utilized for molecular docking analysis. The protein-ligand complexes were performed by AutoDock Vina (<https://autodock.scripps.edu>).

**Supplementary Table 1**. The 20 most upregulated differentially expressed proteins responding to the ratio of the aloin treatment to control.

| **No.** | **Gene** | **Protein Name** | **FC** | **Log_2_FC** | **p value** |
| --- | --- | --- | --- | --- | --- |
| 1 | PKP1 | PKP1 protein | 32 | 5 | 4.18E-12 |
| 2 | YWHAZ | G protein subunit alpha 12 | 32 | 5 | 5.65E-10 |
| 3 | FAM83F | DNA-directed primase/polymerase protein | 32 | 5 | 5.18E-09 |
| 4 | IMPDH2 | MAP3K12-binding inhibitory protein 1 | 32 | 5 | 6.20E-09 |
| 5 | GNA12 | Band 4.1-like protein 3 | 32 | 5 | 7.39E-09 |
| 6 | POLD4 | Protein FAM83F | 32 | 5 | 1.06E-08 |
| 7 | EPB41L3 | Tyrosine 3-monooxygenase | 32 | 5 | 2.60E-08 |
| 8 | MBIP | Cathepsin V | 32 | 5 | 3.74E-08 |
| 9 | TRIB1 | Collagen alpha-2(VI) chain | 32 | 5 | 5.09E-08 |
| 10 | PIGV | Glutathione S-transferase Mu 4 | 32 | 5 | 1.44E-07 |
| 11 | CANT1 | DNA-directed RNA polymerase | 32 | 5 | 1.57E-07 |
| 12 | PRIMPOL | Dickkopf-related protein 1 | 32 | 5 | 1.87E-07 |
| 13 | COL6A2 | Calcium activated nucleotidase 1 | 32 | 5 | 3.88E-07 |
| 14 | POLRMT | Arginase-2, mitochondrial | 32 | 5 | 4.35E-07 |
| 15 | CAMK2N1 | GPI mannosyltransferase 2 | 32 | 5 | 8.60E-07 |
| 16 | CTSV | DNA polymerase delta subunit 4 | 32 | 5 | 9.99E-07 |
| 17 | DKK1 | Keratinocyte proline-rich protein | 32 | 5 | 1.50E-06 |
| 18 | GSTM4 | G1/S-specific cyclin-D3 | 32 | 5 | 4.33E-06 |
| 19 | KPRP | DNA replication factor Cdt1 | 32 | 5 | 8.77E-06 |
| 20 | PKN3 | Serine/threonine-protein kinase N3 | 32 | 5 | 1.87E-04 |

**Supplementary Table 2**. The 20 most downregulated differentially expressed proteins responding to the ratio of the aloin treatment to control.

| **No.** | **Gene** | **Protein Name** | **FC** | **Log_2_FC** | **p value** |
| --- | --- | --- | --- | --- | --- |
| 1 | IPPK | Inositol-pentakisphosphate 2-kinase | 1.00E-05 | -16.61 | 2.26E-12 |
| 2 | GRAMD2A | GRAM domain containing 2A | 1.00E-05 | -16.61 | 4.08E-09 |
| 3 | TEAD3 | Transcriptional enhancer factor TEF-5 | 1.00E-05 | -16.61 | 8.44E-08 |
| 4 | RAP1GAP | RAP1 GTPase activating protein | 1.00E-05 | -16.61 | 4.46E-07 |
| 5 | FOXO3 | Forkhead box protein O3 | 1.00E-05 | -16.61 | 3.22E-05 |
| 6 | DCLRE1C | DNA cross-link repair 1C | 1.00E-05 | -16.61 | 2.68E-07 |
| 7 | DNAAF10 | Dynein axonemal assembly factor 10 | 1.00E-05 | -16.61 | 1.38E-07 |
| 8 | COX10 | Protoheme IX farnesyltransferase, mitochondrial | 1.00E-05 | -16.61 | 2.73E-05 |
| 9 | RAPGEF5 | Rap guanine nucleotide exchange factor 5 isoform 1 | 1.00E-05 | -16.61 | 5.70E-07 |
| 10 | PLK4 | Serine/threonine-protein kinase PLK4 | 1.00E-05 | -16.61 | 5.68E-08 |
| 11 | SNX21 | Sorting nexin family member 21 | 1.00E-05 | -16.61 | 3.34E-06 |
| 12 | ASNSD1 | Asparagine synthetase domain containing 1 | 1.00E-05 | -16.61 | 1.67E-07 |
| 13 | MAP3K1 | Mitogen-activated protein kinase kinase kinase 1 | 1.00E-05 | -16.61 | 7.57E-08 |
| 14 | SPRTN | SprT-like N-terminal domain | 1.00E-05 | -16.61 | 3.29E-08 |
| 15 | EIF2AK3 | Eukaryotic translation initiation factor 2 alpha kinase 3 | 1.00E-05 | -16.61 | 7.18E-06 |
| 16 | FSIP2 | Fibrous sheath-interacting protein 2 | 1.00E-05 | -16.61 | 2.46E-08 |
| 17 | ASB1 | Ankyrin repeat and SOCS box containing 1 | 1.00E-05 | -16.61 | 1.26E-06 |
| 18 | SLC35A3 | Solute carrier family 35 member A3 | 1.00E-05 | -16.61 | 2.70E-05 |
| 19 | ZNF362 | Zinc finger protein 362 | 1.00E-05 | -16.61 | 5.18E-05 |
| 20 | - | DNA polymerase kappa | 1.00E-05 | -16.61 | 9.18E-08 |
